# *N*-(4-Meth­oxy-2-methyl-5-nitro­phen­yl)acetamide

**DOI:** 10.1107/S2414314625004705

**Published:** 2025-06-03

**Authors:** Rao M. Uppu, Frank R. Fronczek

**Affiliations:** a Department of Environmental Toxicology Southern University and A&M College Baton Rouge LA 70813 USA; b Department of Chemistry Louisiana State University,Baton Rouge LA 70803 USA; University of Aberdeen, United Kingdom

**Keywords:** crystal structure, alk­oxy­acetanilides, nonenzymatic biotransformation, per­oxy­nitrite, xenobiotics

## Abstract

In the title compound, C_10_H_12_N_2_O_4_, the four substituents lie out of the phenyl plane by varying degrees. The methyl C atom lies 0.019 (3) Å out of plane, while the meth­oxy O and C atoms lie 0.067 (2) and 0.042 (3) Å out of plane, respectively, with the C—C—O—C torsion angle being 3.3 (2)°. The plane of the nitro group is twisted out of the phenyl plane, forming a dihedral angle of 12.03 (9)° with it. The acetamide substituent is twisted considerably more out of the phenyl plane, forming a dihedral angle of 47.24 (6)° with it. In the extended structure, the acetamide NH group donates a hydrogen bond to an acetamide carbonyl O atom, thereby forming chains propagating in the [010] direction.

## Abstract 2

In the title compound, C_10_H_12_N_2_O_4_, the four substituents lie out of the phenyl plane by varying degrees. In the extended structure, the acetamide NH group donates a hydrogen bond to an acetamide carbonyl O atom, thereby forming chains propagating in the [010] direction.

## Graphical abstract

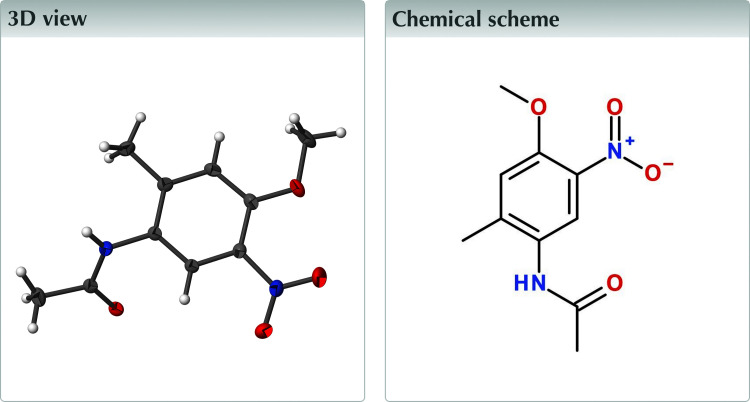

## Structure description

The title compound, C_10_H_12_N_2_O_4_, is a nitro-derivative of 2-meth­ylmethacetin [*N*-(4-meth­oxy-2-meth­ylphen­yl)acetamide]. It is likely formed during per­oxy­nitrite-mediated oxidation of 2-meth­ylmethacetin under physiologically relevant pH and bicarbonate conditions (Hines *et al.*, 2025 ▸). The reaction is consistent with electrophilic nitration initiated by the *in situ* generation of the free-radical oxidants nitro­gen dioxide (^**.**^NO_2_) and carbonate radical (CO_3_^**.**−^) from the inter­action of the per­oxy­nitrite anion (ONOO^−^) with CO_2_ (Agu *et al.*, 2020 ▸; Deere *et al.*, 2020 ▸; Lymar & Hurst, 1995 ▸; Uppu *et al.*, 2000 ▸; Uppu & Pryor, 1996 ▸; Uppu & Pryor, 1999 ▸).

Phenacetin [*N*-(4-eth­oxy­phen­yl)acetamide, C_10_H_13_NO_2_], methacetin [*N*-(4-meth­oxy­phen­yl)acetamide, C_9_H_11_NO_2_] and propacetin [*N*-(4-prop­oxy­phen­yl)acetamide, C_11_H_15_NO_2_] were among the earliest synthetic anti­pyretic–analgesic agents examined in depth (Merck, 1899 ▸). Inter­est in these congeners grew after their precursor acetanilide (Anti­febrin, introduced in 1880) was linked to methemoglobinaemia and cyano­sis owing to excessive formation of its aniline metabolite. Comparative studies in the late 19th and early 20th centuries showed that methacetin possessed the strongest anti­pyretic and analgesic activity, followed by phenacetin and then propacetin, each acting through metabolic release of 4-amino­phenol (Starmer *et al.*, 1971 ▸). While all three function largely as pro-drugs, undergoing rapid oxidative *O*-de­alkyl­ation to produce the active metabolite 4-hy­droxy­acetanilide (Brodie & Axelrod, 1948 ▸; Kapetanović *et al.*, 1979 ▸; Kapetanović & Mieyal, 1979 ▸), a minor *N*-de­acetyl­ation pathway yields 4-alk­oxy­anilines that can be further oxidized to reactive 4-*N*-hy­droxy and 4-nitroso derivatives, leading to methemoglobinaemia, nephrotoxicity and, in the case of phenacetin, urothelial cancer (Prescott, 1980 ▸; Hinson, 1983 ▸). Toxicological studies in experimental animals revealed that methacetin, with the shortest alkyl chain, exhibited higher toxicity, while phenacetin, with a moderate chain length, offered a better balance between efficacy and reduced toxicity (Starmer *et al.*, 1971 ▸). Consequently, phenacetin remained widely used until it was ultimately replaced by acetamino­phen [*N*-(4-hy­droxy­phen­yl)acetamide] in the 1980s as a safer alternative [FDA (Food and Drug Administration), 1983 ▸; IARC (Inter­national Agency for Research on Cancer), 1987 ▸]. There is no evidence that 2-meth­ylmethacetin itself was ever marketed or tested in humans during that era (Merck, 1952 ▸). Recent studies show that oxidative *O*-de­methyl­ation of methacetin-(methyl-^13^C) and subsequent conversion of H^13^CHO to ^13^CO_2_ in LiMAx/MBT breath testing provide broad diagnostic utility across diverse clinical applications (Buechter & Gerken, 2022 ▸; Gairing *et al.*, 2022 ▸; Santol *et al.*, 2024 ▸).

Non-enzymatic oxidation of 4-alk­oxy­acetanilides was largely unexplored until reactive oxygen and nitro­gen species (RONS) were shown to nitrate 4-hy­droxy­acetanilide *in vitro* (Uppu & Martin, 2005 ▸; Deere *et al.*, 2020 ▸). We reasoned that analogous reactions might affect other 4-alk­oxy congeners. Indeed, treating 2-meth­ylmethacetin with per­oxy­nitrite in bicarbonate-enriched buffers at and around neutral pH yielded the title compound as the major product (Hines *et al.*, 2025 ▸). The electron-donating 4-meth­oxy group directs nitration *ortho* to itself, whereas the acetamido group weakly deactivates the ring *ortho* to the amide; consequently, nitration occurs preferentially at C5 to give *N*-(4-meth­oxy-2-methyl-5-nitro­phen­yl)acetamide rather than the C6 position with little or no detectable formation of *N*-(4-meth­oxy-2-methyl-6-nitro­phen­yl)acetamide (Hines *et al.*, 2025 ▸; Uppu & Martin, 2005 ▸). Towards better understanding of the mechanisms of electrophilic nitration of 4-alk­oxy­acetanilides by free radical oxidants formed in per­oxy­nitrite/CO_2_ reactions (Uppu & Pryor, 1999 ▸; Uppu *et al.*, 2000 ▸) and to shed light on mol­ecular targets, we grew crystals of *N*-(4-meth­oxy-2-methyl-5-nitro­phen­yl)acetamide in water and analyzed them by X-ray diffraction.

**Figure 1:**
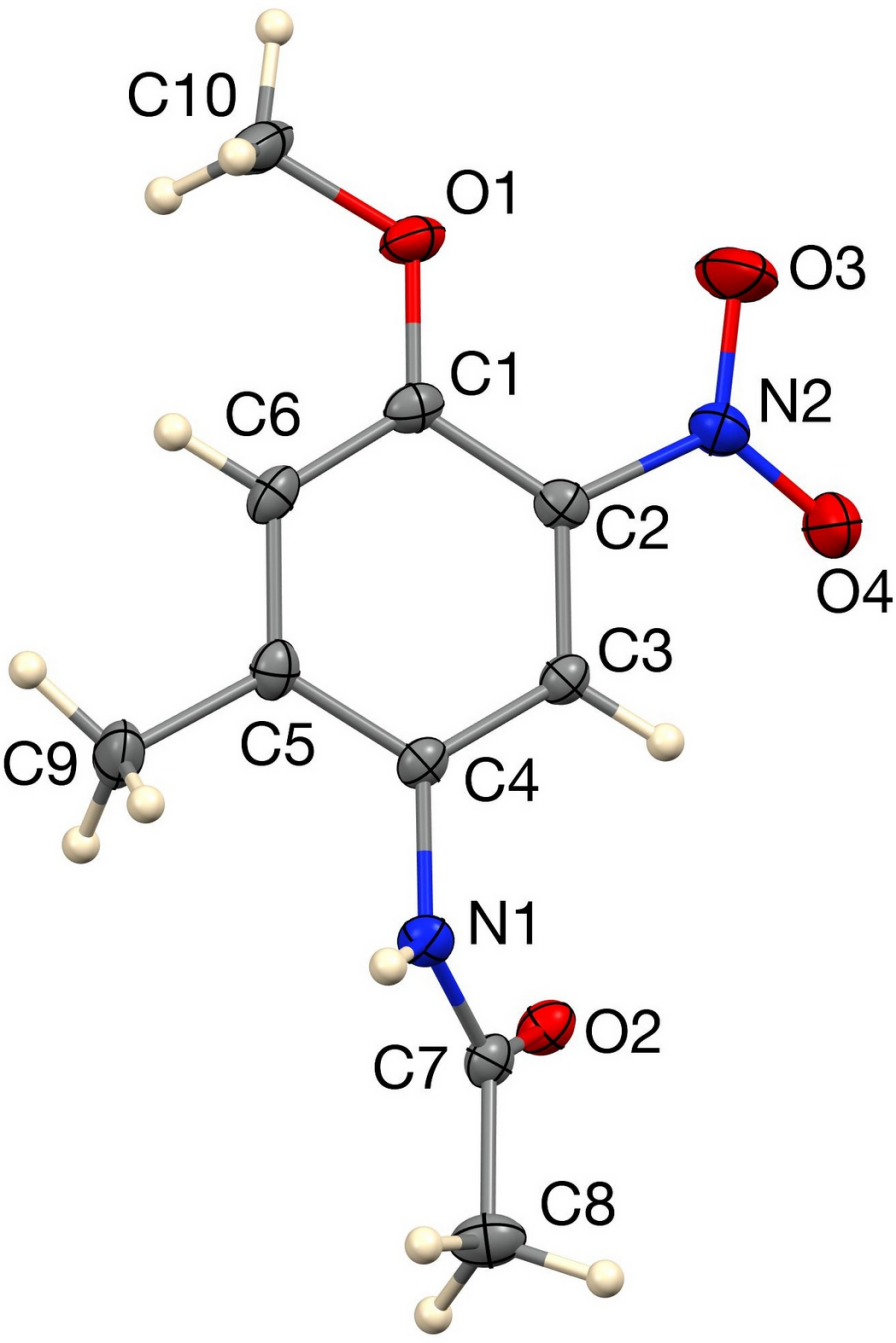
The asymmetric unit of the title compound with 50% probability ellipsoids.

**Figure 2:**
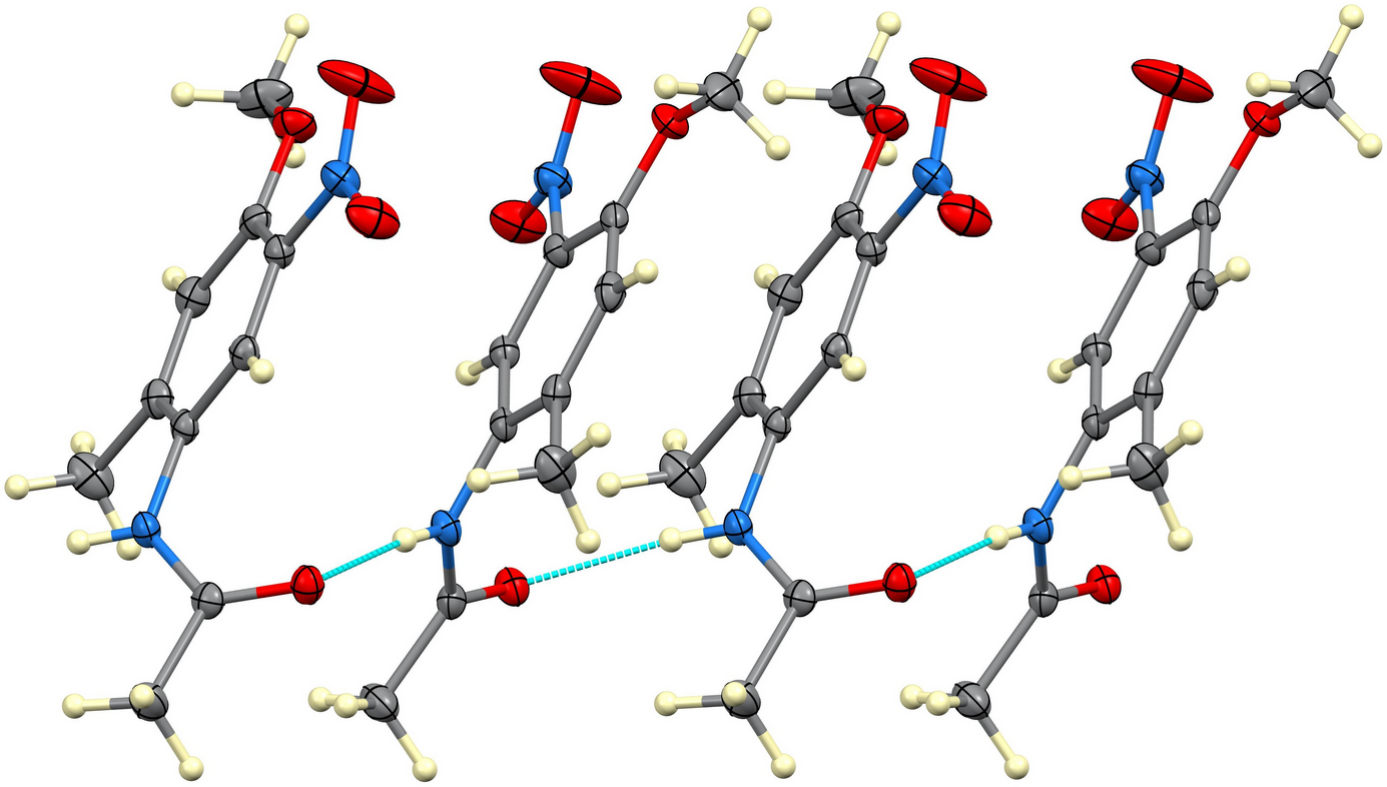
Fragment of a [010] hydrogen-bonded chain.

**Figure 3:**
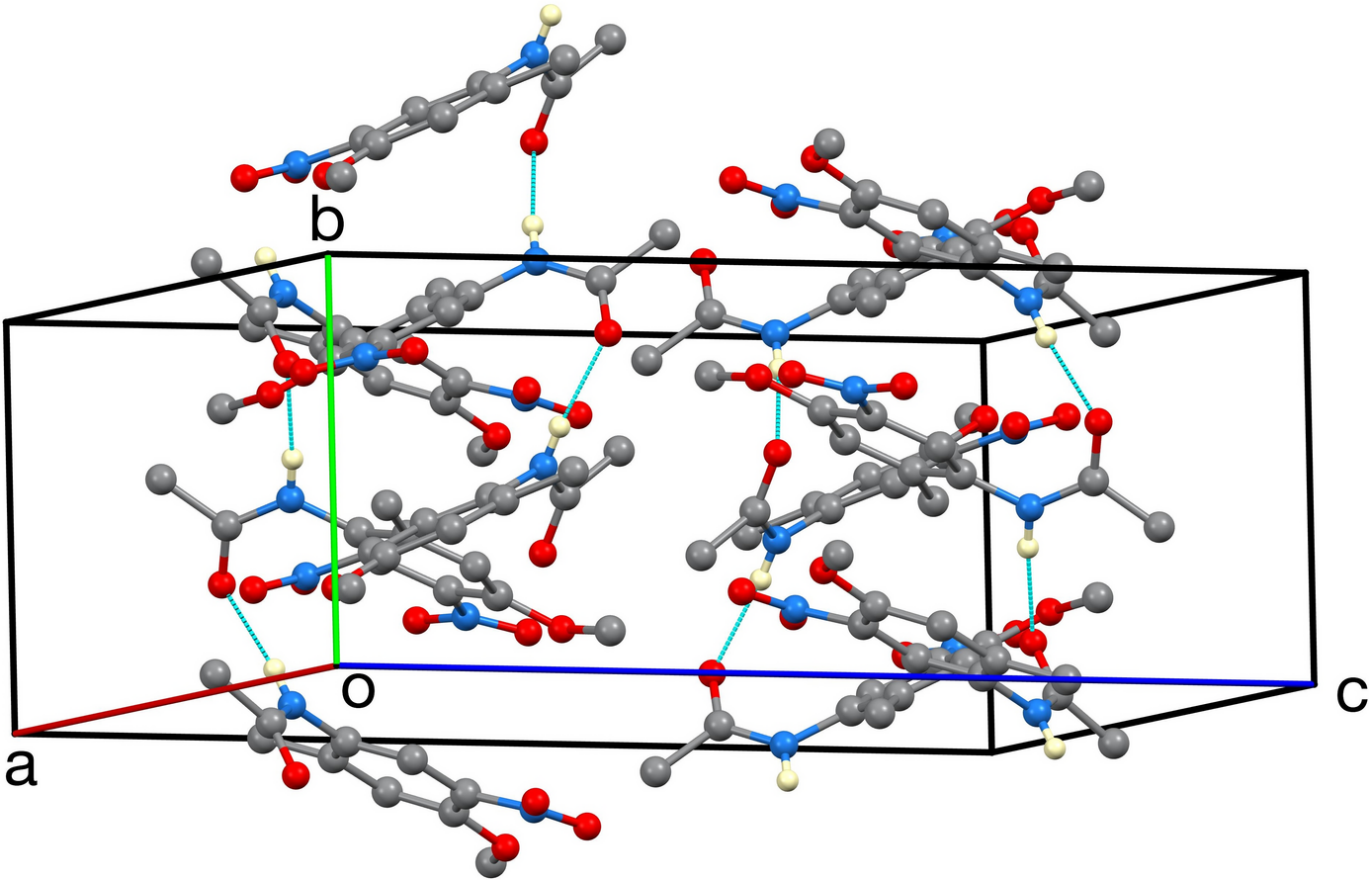
The unit cell. Only N-bound hydrogen atoms are shown.

Single-crystal X-ray diffraction confirms this regiochemistry. The mol­ecular structure (Fig. 1 ▸) shows the C1–C6 aromatic ring, meth­oxy group (C1–O1–C10H_3_) and C9 methyl carbon atom to be nearly coplanar (r.m.s. deviation = 0.012 Å), whereas the N2/O3/O4 nitro group at C2 is twisted about the C2—N2 bond such that the plane of the nitro group forms a dihedral angle of 12.03 (9)° with the phenyl plane. The N1/C7/C8/O2 acetamide substituent is twisted considerably more out of the phenyl plane, forming a dihedral angle of 47.24 (6)° with it. In the crystal, N1—H1*N*⋯O2(carbonyl) hydrogen bonds [N⋯O = 2.8636 (16) Å] assemble the mol­ecules into [010] chains (Table 1 ▸, Fig. 2 ▸); weaker C—H⋯O contacts link these chains into layers, giving the overall packing illustrated in Fig. 3 ▸. The 2-methyl substituent takes no part in specific inter­molecular inter­actions but influences packing through van der Waals contacts.

The structure of the title compound represents the first crystallographic characterization of a nitrated 4-alk­oxy­acetanilide formed under biomimetic RONS conditions. Its isolation in per­oxy­nitrite/CO_2_-mediated oxidation of *N*-(4-meth­oxy-2-meth­yl)acetamide strongly suggests the possibility that analogous nitrated metabolites may arise *in vivo* during oxidative stress, potentially modulating the pharmacology or toxicity of 4-alk­oxy­acetanilide analgesics.

In terms of mol­ecular planarity and substituent orientations, *N*-(4-meth­oxy-2-nitro­phen­yl)acetamide (Hines *et al.*, 2022 ▸), *N*-(4-meth­oxy-3-nitro­phen­yl)acetamide (Hines *et al.*, 2023 ▸) and the title compound share a benzene ring with the *para*-meth­oxy group nearly coplanar to it (C—C—O—C torsion angles on the order of 0–6°). Significant differences emerge in the disposition of the nitro and acetamide substituents. For instance, in *N*-(4-meth­oxy-3-nitro­phen­yl)acetamide, the acetamide moiety lies essentially in the aromatic plane (the C—N—C=O dihedral angle is close to 0°), making the entire meth­oxy­phenyl-acetamide fragment nearly planar (r.m.s. deviation ∼0.04 Å). The nitro group at the *meta* position is rotated out of the ring plane (∼30°) and is disordered over two orientations. In *N*-(4-meth­oxy-3-nitro­phen­yl)acetamide and *N*-(4-meth­oxy-2-methyl-5-nitro­phen­yl)acetamide, with the nitro substituent *ortho* to the anilide nitro­gen atom, the phenyl and acetamide groups are not coplanar. The acetamide group is tilted by about 25° in *N*-(4-meth­oxy-2-nitro­phen­yl)acetamide and as much as ∼47° in *N*-(4-meth­oxy-2-methyl-5-nitro­phen­yl)acetamide, due to steric inter­ference from the *ortho* substituents. Meanwhile, their nitro groups (designated as either 2- or 5-position on the ring) are only moderately twisted out of the plane (on the order of 12°), a considerably smaller deviation than in *N*-(4-meth­oxy-3-nitro­phen­yl)acetamide.

Regarding inter­molecular inter­actions, the presence or absence of an *ortho* nitro group governs the hydrogen-bonding patterns. In the 2-nitro compound [*N*-(4-meth­oxy-2-nitro­phen­yl)acetamide] (Hines *et al.*, 2022 ▸), an intra­molecular N—H⋯O hydrogen bond links the amide N—H group to the *ortho* nitro oxygen atom. This inter­nal hydrogen bond satisfies the donor, so no strong inter­molecular N—H bonds occur; instead, packing is consolidated by weaker contacts (*e.g*., a C—H⋯O contact between mol­ecules) and exhibits a herringbone motif (adjacent phenyl rings are inclined by ∼65° rather than stacked parallel). In the 3-nitro isomer [*N*-4-meth­oxy-3-nitro­phen­yl)acetamide] (Hines *et al.*, 2023 ▸), in contrast, there is no provision for an intra­molecular hydrogen bond. Accordingly, each N—H group donates to a nitro oxygen atom on a neighboring mol­ecule, forming N—H⋯O(nitro) chains in the crystal (the amide carbonyl O is not an acceptor in this structure). The 2-methyl-5-nitro derivative lacks an *ortho* nitro acceptor, and it instead exhibits the conventional amide catemer: the N—H hydrogen bonds to the carbonyl O atom of an adjacent mol­ecule, linking mol­ecules into N—H⋯O=C chains propagating through the structure. Importantly, none of nitro derivatives shows significant π–π stacking between aromatic rings; for example, the 2-nitro and 2-methyl-5-nitro crystals adopt a crossed herringbone-like packing rather than face-to-face stacks.

## Synthesis and crystallization

*N-*(4-Meth­oxy-2-methyl-5-nitro­phen­yl)acetamide (CAS 196194–97-5), was obtained from AmBeed (Arlington Heights, Illinois, USA) and was used without further purification. Crystals in the form of colorless laths were prepared by slow cooling of a nearly saturated solution of the title compound in boiling deionized water (resistance *ca*. 18 *M*Ω.cm^−1^).

## Refinement

Crystal data, data collection and structure refinement details are summarized in Table 2 ▸.

## Supplementary Material

Crystal structure: contains datablock(s) I. DOI: 10.1107/S2414314625004705/hb4520sup1.cif

Structure factors: contains datablock(s) I. DOI: 10.1107/S2414314625004705/hb4520Isup2.hkl

Supporting information file. DOI: 10.1107/S2414314625004705/hb4520Isup3.cml

CCDC reference: 2453807

Additional supporting information:  crystallographic information; 3D view; checkCIF report

## Figures and Tables

**Table 1:** Hydrogen-bond geometry (Å, °)

*D*—H⋯*A*	*D*—H	H⋯*A*	*D*⋯*A*	*D*—H⋯*A*
N1—H1*N*⋯O2^i^	0.84 (2)	2.03 (2)	2.8636 (16)	174 (2)
C9—H9*A*⋯O2^ii^	0.98	2.54	3.5009 (19)	166
C9—H9*B*⋯O2^i^	0.98	2.47	3.2971 (19)	142

Symmetry codes: (i) 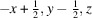; (ii) 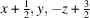.

**Table 2:** Experimental details

Crystal data	
Chemical formula	C_10_H_12_N_2_O_4_
*M*_r_	224.22
Crystal system, space group	Orthorhombic, *Pbca*
Temperature (K)	100
*a*, *b*, *c* (Å)	14.2323 (6), 7.6198 (3), 19.8463 (8)
*V* (Å^3^)	2152.28 (15)
*Z*	8
Radiation type	Cu *K*α
μ (mm^−1^)	0.92
Crystal size (mm)	0.23 × 0.04 × 0.02

Data collection	
Diffractometer	Bruker D8 Venture DUO with Photon III C14
Absorption correction	Multi-scan (*SADABS*; Krause *et al.*, 2015 ▸)
*T*_min_, *T*_max_	0.722, 0.982
No. of measured, independent and observed [*I* > 2σ(*I*)] reflections	25336, 2301, 1853
*R*_int_	0.160
(sin θ/λ)_max_ (Å^−1^)	0.638

Refinement	
*R*[*F*^2^ > 2σ(*F*^2^)], *wR*(*F*^2^), *S*	0.053, 0.136, 1.04
No. of reflections	2301
No. of parameters	151
H-atom treatment	H atoms treated by a mixture of independent and constrained refinement
Δρ_max_, Δρ_min_ (e Å^−3^)	0.37, −0.41

Computer programs: *APEX5* and *SAINT* (Bruker, 2016 ▸), *SHELXT2018/2* (Sheldrick, 2015*a* ▸), *SHELXL2019/1* (Sheldrick, 2015*b* ▸), *Mercury* (Macrae *et al.*, 2020 ▸), *publCIF* (Westrip, 2010 ▸).

## full crystallographic data

### N-(4-Methoxy-2-methyl-5-nitrophenyl)acetamide Crystal data

**Table:**

C_10_H_12_N_2_O_4_	*D*_x_ = 1.384 Mg m^−^^3^
*M**_r_* = 224.22	Cu *K*α radiation, λ = 1.54184 Å
Orthorhombic, *P**b**c**a*	Cell parameters from 8628 reflections
*a* = 14.2323 (6) Å	θ = 4.5–79.4°
*b* = 7.6198 (3) Å	µ = 0.92 mm^−^^1^
*c* = 19.8463 (8) Å	*T* = 100 K
*V* = 2152.28 (15) Å^3^	Lath, colourless
*Z* = 8	0.23 × 0.04 × 0.02 mm
*F*(000) = 944	

### N-(4-Methoxy-2-methyl-5-nitrophenyl)acetamide Data collection

**Table:**

Bruker D8 Venture DUO with Photon III C14 diffractometer	1853 reflections with *I* > 2σ(*I*)
Radiation source: IµS 3.0 microfocus	*R*_int_ = 0.160
φ and ω scans	θ_max_ = 79.5°, θ_min_ = 4.5°
Absorption correction: multi-scan (SADABS; Krause *et al.*, 2015)	*h* = −18→16
*T*_min_ = 0.722, *T*_max_ = 0.982	*k* = −9→9
25336 measured reflections	*l* = −24→25
2301 independent reflections	

### N-(4-Methoxy-2-methyl-5-nitrophenyl)acetamide Refinement

**Table:**

Refinement on *F*^2^	Primary atom site location: dual
Least-squares matrix: full	Hydrogen site location: mixed
*R*[*F*^2^ > 2σ(*F*^2^)] = 0.053	H atoms treated by a mixture of independent and constrained refinement
*wR*(*F*^2^) = 0.136	*w* = 1/[σ^2^(*F*_o_^2^) + (0.0839*P*)^2^ + 0.5117*P*] where *P* = (*F*_o_^2^ + 2*F*_c_^2^)/3
*S* = 1.04	(Δ/σ)_max_ = 0.001
2301 reflections	Δρ_max_ = 0.37 e Å^−^^3^
151 parameters	Δρ_min_ = −0.41 e Å^−^^3^
0 restraints	

### N-(4-Methoxy-2-methyl-5-nitrophenyl)acetamide Special details

**Table:**

Geometry. All esds (except the esd in the dihedral angle between two l.s. planes) are estimated using the full covariance matrix. The cell esds are taken into account individually in the estimation of esds in distances, angles and torsion angles; correlations between esds in cell parameters are only used when they are defined by crystal symmetry. An approximate (isotropic) treatment of cell esds is used for estimating esds involving l.s. planes.
Refinement. All H atoms were located in difference maps and those on C were thereafter treated as riding in geometrically idealized positions with C—H distances 0.95 Å for phenyl and 0.98 Å for methyl. Coordinates of the N—H atom were refined. *U*_iso_(H) values were assigned as 1.2*U*_eq_ for the attached atom (1.5 for methyl). Torsional parameters were refined for the methyl groups.

### N-(4-Methoxy-2-methyl-5-nitrophenyl)acetamide Fractional atomic coordinates and isotropic or equivalent isotropic displacement parameters (Å^2^)

**Table:**

	*x*	*y*	*z*	*U*_iso_*/*U*_eq_	
O1	0.34418 (8)	0.78233 (14)	0.55179 (6)	0.0238 (3)	
O2	0.14170 (7)	0.65326 (13)	0.83184 (5)	0.0199 (3)	
O3	0.16525 (11)	0.7593 (3)	0.52701 (8)	0.0556 (5)	
O4	0.07202 (8)	0.71866 (19)	0.61014 (6)	0.0326 (3)	
N1	0.25448 (9)	0.46222 (15)	0.79712 (6)	0.0173 (3)	
H1N	0.2850 (15)	0.370 (3)	0.8043 (11)	0.021*	
N2	0.15115 (9)	0.72149 (17)	0.58592 (7)	0.0222 (3)	
C1	0.32443 (10)	0.70066 (17)	0.61038 (7)	0.0177 (3)	
C2	0.23007 (10)	0.67361 (18)	0.62915 (7)	0.0169 (3)	
C3	0.20741 (9)	0.59707 (17)	0.69062 (7)	0.0159 (3)	
H3	0.143309	0.580561	0.702360	0.019*	
C4	0.27693 (10)	0.54469 (17)	0.73487 (7)	0.0154 (3)	
C5	0.37149 (10)	0.56655 (18)	0.71717 (7)	0.0181 (3)	
C6	0.39333 (10)	0.64377 (18)	0.65543 (8)	0.0192 (3)	
H6	0.457538	0.658219	0.643570	0.023*	
C7	0.18799 (10)	0.51835 (18)	0.84076 (7)	0.0174 (3)	
C8	0.17255 (12)	0.4061 (2)	0.90199 (8)	0.0263 (3)	
H8A	0.221118	0.315107	0.904041	0.039*	
H8B	0.110504	0.350799	0.899344	0.039*	
H8C	0.175971	0.479260	0.942536	0.039*	
C9	0.44939 (11)	0.5085 (2)	0.76307 (9)	0.0274 (4)	
H9A	0.510092	0.538858	0.742883	0.041*	
H9B	0.445817	0.381196	0.769593	0.041*	
H9C	0.443105	0.567589	0.806702	0.041*	
C10	0.44162 (13)	0.8017 (3)	0.53463 (8)	0.0313 (4)	
H10A	0.447058	0.860973	0.490986	0.047*	
H10B	0.471040	0.685593	0.531880	0.047*	
H10C	0.473279	0.871596	0.569293	0.047*	

### N-(4-Methoxy-2-methyl-5-nitrophenyl)acetamide Atomic displacement parameters (Å^2^)

**Table:**

	*U*^11^	*U*^22^	*U*^33^	*U*^12^	*U*^13^	*U*^23^
O1	0.0276 (6)	0.0242 (6)	0.0196 (5)	−0.0065 (4)	0.0059 (4)	0.0035 (4)
O2	0.0197 (5)	0.0166 (5)	0.0235 (5)	0.0003 (4)	0.0032 (4)	0.0017 (4)
O3	0.0339 (7)	0.1045 (14)	0.0285 (7)	0.0073 (8)	0.0004 (5)	0.0335 (8)
O4	0.0196 (6)	0.0494 (8)	0.0288 (6)	0.0028 (5)	−0.0025 (4)	0.0087 (5)
N1	0.0186 (6)	0.0131 (6)	0.0201 (6)	0.0017 (4)	0.0001 (4)	0.0031 (4)
N2	0.0238 (6)	0.0227 (6)	0.0203 (6)	0.0004 (5)	−0.0014 (5)	0.0045 (5)
C1	0.0230 (7)	0.0121 (6)	0.0182 (6)	−0.0033 (5)	0.0036 (5)	−0.0009 (5)
C2	0.0192 (7)	0.0138 (6)	0.0177 (6)	0.0008 (5)	−0.0007 (5)	0.0009 (5)
C3	0.0162 (6)	0.0130 (6)	0.0185 (6)	−0.0010 (5)	0.0015 (5)	0.0002 (5)
C4	0.0171 (7)	0.0105 (6)	0.0185 (7)	−0.0014 (5)	0.0011 (5)	0.0002 (4)
C5	0.0172 (7)	0.0136 (6)	0.0235 (7)	−0.0025 (5)	−0.0011 (5)	−0.0006 (5)
C6	0.0161 (6)	0.0165 (6)	0.0251 (7)	−0.0035 (5)	0.0033 (5)	−0.0015 (5)
C7	0.0180 (6)	0.0144 (6)	0.0198 (6)	−0.0036 (5)	−0.0002 (5)	−0.0001 (5)
C8	0.0339 (8)	0.0213 (7)	0.0236 (7)	0.0013 (6)	0.0056 (6)	0.0052 (6)
C9	0.0170 (7)	0.0314 (8)	0.0340 (8)	−0.0035 (6)	−0.0047 (6)	0.0080 (6)
C10	0.0318 (9)	0.0375 (9)	0.0245 (7)	−0.0168 (7)	0.0098 (6)	−0.0017 (6)

### N-(4-Methoxy-2-methyl-5-nitrophenyl)acetamide Geometric parameters (Å, º)

**Table:**

O1—C1	1.3485 (17)	C4—C5	1.4009 (19)
O1—C10	1.436 (2)	C5—C6	1.394 (2)
O2—C7	1.2338 (18)	C5—C9	1.502 (2)
O3—N2	1.221 (2)	C6—H6	0.9500
O4—N2	1.225 (2)	C7—C8	1.502 (2)
N1—C7	1.3522 (19)	C8—H8A	0.9800
N1—C4	1.4224 (17)	C8—H8B	0.9800
N1—H1N	0.84 (2)	C8—H8C	0.9800
N2—C2	1.4597 (19)	C9—H9A	0.9800
C1—C6	1.396 (2)	C9—H9B	0.9800
C1—C2	1.409 (2)	C9—H9C	0.9800
C2—C3	1.3902 (19)	C10—H10A	0.9800
C3—C4	1.3818 (19)	C10—H10B	0.9800
C3—H3	0.9500	C10—H10C	0.9800
			
C1—O1—C10	116.96 (13)	C5—C6—H6	118.8
C7—N1—C4	125.01 (12)	C1—C6—H6	118.8
C7—N1—H1N	121.3 (15)	O2—C7—N1	123.05 (13)
C4—N1—H1N	113.7 (15)	O2—C7—C8	120.82 (13)
O3—N2—O4	122.09 (14)	N1—C7—C8	116.13 (13)
O3—N2—C2	119.70 (14)	C7—C8—H8A	109.5
O4—N2—C2	118.20 (13)	C7—C8—H8B	109.5
O1—C1—C6	123.30 (13)	H8A—C8—H8B	109.5
O1—C1—C2	119.62 (14)	C7—C8—H8C	109.5
C6—C1—C2	117.06 (13)	H8A—C8—H8C	109.5
C3—C2—C1	120.96 (13)	H8B—C8—H8C	109.5
C3—C2—N2	116.25 (12)	C5—C9—H9A	109.5
C1—C2—N2	122.79 (13)	C5—C9—H9B	109.5
C4—C3—C2	120.86 (13)	H9A—C9—H9B	109.5
C4—C3—H3	119.6	C5—C9—H9C	109.5
C2—C3—H3	119.6	H9A—C9—H9C	109.5
C3—C4—C5	119.62 (13)	H9B—C9—H9C	109.5
C3—C4—N1	121.25 (12)	O1—C10—H10A	109.5
C5—C4—N1	119.08 (13)	O1—C10—H10B	109.5
C6—C5—C4	118.99 (13)	H10A—C10—H10B	109.5
C6—C5—C9	119.53 (13)	O1—C10—H10C	109.5
C4—C5—C9	121.48 (13)	H10A—C10—H10C	109.5
C5—C6—C1	122.49 (13)	H10B—C10—H10C	109.5
			
C10—O1—C1—C6	3.3 (2)	C2—C3—C4—N1	−178.38 (12)
C10—O1—C1—C2	−178.34 (13)	C7—N1—C4—C3	−46.4 (2)
O1—C1—C2—C3	−177.04 (12)	C7—N1—C4—C5	136.18 (15)
C6—C1—C2—C3	1.4 (2)	C3—C4—C5—C6	1.1 (2)
O1—C1—C2—N2	3.6 (2)	N1—C4—C5—C6	178.54 (12)
C6—C1—C2—N2	−177.98 (13)	C3—C4—C5—C9	−178.84 (14)
O3—N2—C2—C3	−166.83 (17)	N1—C4—C5—C9	−1.4 (2)
O4—N2—C2—C3	11.7 (2)	C4—C5—C6—C1	0.1 (2)
O3—N2—C2—C1	12.6 (2)	C9—C5—C6—C1	179.99 (14)
O4—N2—C2—C1	−168.90 (14)	O1—C1—C6—C5	177.07 (13)
C1—C2—C3—C4	−0.3 (2)	C2—C1—C6—C5	−1.3 (2)
N2—C2—C3—C4	179.11 (12)	C4—N1—C7—O2	−2.9 (2)
C2—C3—C4—C5	−1.0 (2)	C4—N1—C7—C8	177.03 (13)

### N-(4-Methoxy-2-methyl-5-nitrophenyl)acetamide Hydrogen-bond geometry (Å, º)

**Table:**

*D*—H···*A*	*D*—H	H···*A*	*D*···*A*	*D*—H···*A*
N1—H1*N*···O2^i^	0.84 (2)	2.03 (2)	2.8636 (16)	174 (2)
C9—H9*A*···O2^ii^	0.98	2.54	3.5009 (19)	166
C9—H9*B*···O2^i^	0.98	2.47	3.2971 (19)	142

Symmetry codes: (i) −*x*+1/2, *y*−1/2, *z*; (ii) *x*+1/2, *y*, −*z*+3/2.
